# The effectiveness of medical assistant health coaching for low-income patients with uncontrolled diabetes, hypertension, and hyperlipidemia: protocol for a randomized controlled trial and baseline characteristics of the study population

**DOI:** 10.1186/1471-2296-14-27

**Published:** 2013-02-23

**Authors:** Rachel Willard-Grace, Denise DeVore, Ellen H Chen, Danielle Hessler, Thomas Bodenheimer, David H Thom

**Affiliations:** 1 Department of Family and Community Medicine, University of California San Francisco, San Francisco, CA, USA; 2 San Francisco Department of Public Health, Silver Avenue Family Health Center, San Francisco, CA, USA

**Keywords:** Self-management support, Chronic disease, Health coach, Patient care team, Diabetes, Hypertension, Hyperlipidemia, Safety net, Primary care

## Abstract

**Background:**

Many patients with chronic disease do not reach goals for management of their conditions. Self-management support provided by medical assistant health coaches within the clinical setting may help to improve clinical outcomes, but most studies to date lack statistical power or methodological rigor. Barriers to large scale implementation of the medical assistant coach model include lack of clinician buy-in and the absence of a business model that will make medical assistant health coaching sustainable. This study will add to the evidence base by determining the effectiveness of health coaching by medical assistants on clinical outcomes and patient self-management, by assessing the impact of health coaching on the clinician experience, and by examining the costs and potential savings of health coaching.

**Methods/Design:**

This randomized controlled trial will evaluate the effectiveness of clinic-based medical assistant health coaches to improve clinical outcomes and self-management skills among low-income patients with uncontrolled type 2 diabetes, hypertension, or hyperlipidemia. A total of 441 patients from two San Francisco primary care clinics have been enrolled and randomized to receive a health coach (n = 224) or usual care (n = 217). Patients participating in the health coaching group will receive coaching for 12 months from medical assistants trained as health coaches. The primary outcome is a change in hemoglobin A1c, systolic blood pressure, or LDL cholesterol among patients with uncontrolled diabetes, hypertension and hyperlipidemia, respectively. Self-management behaviors, perceptions of the health care team and clinician, BMI, and chronic disease self-efficacy will be measured at baseline and after 12 months. Clinician experience is being assessed through surveys and qualitative interviews. Cost and utilization data will be analyzed through cost-predictive models.

**Discussion:**

Medical assistants are an untapped resource to provide self-management support for patients with uncontrolled chronic disease. Having successfully completed recruitment, this study is uniquely poised to assess the effectiveness of the medical assistant health coaching model, to describe barriers and facilitators to implementation, and to develop a business case for sustainability.

**Trial registration:**

ClinicalTrials.gov identifier NCT-01220336

## Background

Chronic disease accounts for more than 80% of health care spending in the United States. Diabetes costs $132 billion each year in medical expenditures, lost workdays, and permanent disability [1] and is projected to reach $192 billion in 2020 [2]. Cardiovascular disease costs $394 billion annually [1].

Medication adherence and lifestyle changes coupled with evidence-based practice guidelines are effective tools to control chronic disease. Yet half of patients with hypertension, 43% of people with diabetes, and 80% of people with hyperlipidemia have not reached their respective goals for blood pressure, glycemic control, or lipids [3-5]. Half of patients do not take their chronic disease medications as prescribed, and only one in ten patients follow recommended guidelines for lifestyle changes, such as smoking cessation or healthy eating [6]. Minority and low-income communities bear a disproportionate burden of chronic disease and its complications [7], and they are less likely to engage in effective self-management of their conditions [8,9].

Traditional didactic education shows little correlation with clinical outcomes such as glycemic control, blood pressure, and cholesterol [10]. In contrast, self-management support, defined by the Institute of Medicine as the “systematic provision of education and supportive interventions to increase patients' skills and confidence in managing their health conditions,” has been shown to improve clinical outcomes [11-13]. Health Coaching, which is one form of self-management support, is designed to empower patients within the health care setting and in their daily lives [14]. Within the health care setting, empowerment is characterized by voicing concerns, asking questions, providing information about home monitoring, and collaboratively developing care plans. In their daily lives, empowered patients are more likely to adhere to treatment plans and engage in lifestyle changes to effectively manage their chronic conditions [15,16].

There is growing evidence that primary care clinicians (physicians, nurse practitioners, and physician assistants) are not able to provide all needed preventive and chronic care support alone. It would require an estimated 21.7 hours per day for a clinician to meet the chronic, preventive, and acute care needs of a panel of 2,500 patients [17,18]. New evidence-based models of care are needed to provide self-management support in primary care that is culturally and linguistically appropriate, as well as financially sustainable in resource-poor settings. Various members of the health care team have been proposed to deliver self-management support, such as nurse practitioners [19], registered nurses (RNs) [20,21], medical assistants (MAs) [21-27], volunteers [28], and other patients with the same condition [20,29,30]. Of these, medical assistants represent a uniquely untapped resource for self-management support. As one of the fastest growing allied health professions [31], the medical assistant workforce is more ethnically and linguistically diverse than other medical professions and therefore more culturally and linguistically concordant with patient populations [32]. Moreover, qualitative research on medical assistants has found that they often conceptualize their role as patient liaisons, cultural brokers, and “workers who care,” roles that segue naturally into health coaching [33].

Previous studies of medical assistant health coaching programs found positive trends in clinical outcomes such as hemoglobin A1c but lacked power to find statistically significant differences [25] or were not designed as randomized trials [22-24].

This is the first large, randomized controlled trial known to the authors to examine the effectiveness of training medical assistants to act as health coaches within primary care practices for patients with uncontrolled type 2 diabetes, hypertension, and hyperlipidemia. The results of this study will provide evidence about the clinical efficacy, barriers and facilitators to implementation, and cost of a health coaching model delivered by medical assistants within the primary care setting.

## Methods/Design

### Study design

The health coaching in primary care (HCPC) study is a two-site, two-armed randomized controlled trial. Randomization was unblinded. The recruitment target was 440 patients enrolled into the study; the study team reached 100% of their recruitment target in April 2012, with 441 patients recruited into the study. The study protocol and materials were approved by the UCSF Committee on Human Research (Approval number: 10–02813), and the study was registered with clinicaltrials.gov (NCT-01220336).

### Setting

The study is being carried out at two sites within the safety net of primary care clinics that serve San Francisco’s low-income or uninsured population. These sites differ in patient demographics, payment mix, and team structure, providing an opportunity for the study to examine implementation facilitators and challenges.

Clinic A is a not-for-profit federally qualified healthcare center. It serves a predominantly Latino population (89% in 2011). The majority of Clinic A’s Adult patients (79% in 2011) are uninsured. In the adult medicine department of the clinic, where this study was conducted, medical assistants are not paired with clinicians in teamlets, instead working with different clinicians every day. The clinic has an integrated behavioral health program, in which clinicians may refer patients to behaviorists, who keep part of their schedule open so as to be able to receive new referrals through face-to-face introductions. Clinic A has two full-time nutritionists/diabetes educators that work with some of the clinic’s diabetic patients. Ten months after the study began, Clinic A “went live” with their electronic medical record (NextGen), which had significant impacts on the clinic flow and acquisition of study information.

Clinic B is a public primary care clinic operated by the San Francisco Department of Public Health. The patient population of the clinic is predominantly African American (64%). Most of the patient population has MediCal (42%) or Healthy San Francisco (31%), a local form of healthcare access for the uninsured. Personnel of the clinic are organized into teamlets, in which each clinician works with the same medical assistant each day. Clinic B also has an integrated behavioral health system and a registered nurse who provides chronic disease education.

### Participants

In order to be eligible for the study, patients must have at least one medical visit to the study site within the last 12 months. They must intend to continue coming to the clinic for the next year with no planned absences of more than four months, and they must have a phone number at which they may be reached. Eligible participants are between 18 and 75 years of age (inclusive) and proficient in English or Spanish. Moreover, they must qualify for the study based on clinical measures indicating diabetes, hypertension, or hyperlipidemia that is uncontrolled at baseline. For the purposes of the study, patients are defined as being diabetic based on a confirmed diagnosis by a medical clinician or at least one prior hemoglobin A1c (HbA1c) ≥6.5.

Exclusion criteria include a diagnosis of type 1 diabetes mellitus; severe or terminal health conditions that would make it inappropriate to focus on improving control of other chronic diseases; or a behavioral health issue that could make it difficult to work with a health coach (e.g., uncontrolled schizophrenia).

Health coaches are required to have a diploma from a medical assistant program (a 3–12 month program) and be bilingual in English and Spanish. None of the three medical assistants selected for the study have attended a four-year college. All three medical assistants selected are female and self-identify as Latina.

### Identification and recruitment

Patients potentially eligible are identified through registry software (i2i tracks). Patients are considered to be potentially eligible if (1) they carry a diagnosis of diabetes and have an HbA1c ≥8.0% within the last year or have not had their HbA1c measured in the past 12 months; (2) if their most recent systolic blood pressure (SBP) is ≥140 mmHg and is within the past 12 months; or (3) if they have calculated low-density lipoprotein (LDL) ≥ 160 (or ≥100 if diabetic) within the last year or have not had their LDL measured in the past 12 months.

Clinicians at the sites receive lists of their patients who are eligible or possibly eligible and are asked to indicate any that should be excluded from the study because of severe or terminal health conditions, behavioral health issues that precludes working with a health coach, or another reason (e.g., patient does not have phone or does not speak English or Spanish).

Patients who are not excluded from the study by their clinician are sent a letter from the clinician introducing the study. The letter provides a number to call if patients do not want to be contacted for the study. Research assistants call these patients at least two weeks after the letters are mailed to invite them to participate in the study and to set up a time to meet.

Potentially eligible patients without recent laboratory testing are contacted and offered the opportunity to have their HbA1c, LDL, and/or SBP measured, after which their eligibility is determined. Patients are considered eligible based on meeting at least one of three clinical criteria: (1) uncontrolled diabetes if they have a HbA1c ≥ 8.0% in the last three months; (2) uncontrolled hypertension if they have a systolic blood pressure of ≥140 at the time of baseline and at the previous visit at least two weeks but not more than a year prior to baseline; or (3) uncontrolled hyperlipidemia if they have an LDL ≥ 160 mg/dl (if not diabetic) or ≥100 (if diabetic) within the past 6 months. If triglycerides are ≤40 or ≥400 mg/dl then non-HDL cholesterol (total cholesterol-HDL) is used with thresholds of ≥190 and ≥130 for non-diabetic and diabetic patients, respectively [34].

### Enrollment and randomization

Research assistants (RAs) meet with eligible patients to explain the study and administer consent and to obtain permission to view the patient’s medical record. If patients give their consent, the RAs conduct a 45-minute verbal survey and measure height and weight. Data is collected electronically using a Microsoft Access database. When the survey is complete, the RAs give the patient a sealed envelope with a randomization card inside that indicates whether the patient is assigned to the health coaching or usual care arm of the study. In the event that the randomization card assigns the patient to receive a health coach, the RA immediately introduces the patient to the health coach. Participants receive $10 for the baseline survey and $10 for the 12-month survey in recognition of the time spent meeting with RAs to take part in the surveys.

### Intervention

Health coaches attend 40 hours of training over six weeks using a curriculum developed by the study team. The curriculum includes instruction in using active listening and non-judgmental communication; helping with self-management skills for diabetes, hypertension, and hyperlipidemia; providing social and emotional support; assisting with lifestyle change; facilitating medication understanding and adherence; navigating the clinic; and accessing community resources. A description of the curriculum can be found at http://familymedicine.medschool.ucsf.edu/cepc/.

The health coach briefly meets with patients assigned to the coaching arm at the time of randomization to explain her role and the ways in which she can support the patient, and she schedules a time to meet with the patient prior to his/her next medical visit. Interactions between health coaches and patients are of three types: medical visits, individual visits, and phone calls. The minimum required frequency of contacts is once every three months for in-person visits (often as part of a medical visit) and monthly for additional contacts such as phone calls.

Medical visits with a health coach consist of a pre-visit, a medical visit, and a post-visit [35]. During the pre-visit, the health coach meets with the patient for medication reconciliation, agenda-setting, and reviewing lab numbers. Medication reconciliation is reviewing current medications to determine whether they are being taken as prescribed, assessing patient knowledge about the purpose of their medications, and identifying and addressing barriers to medication adherence. Agenda setting entails identifying all of the issues of concern to the patient, determining which of these issues are of highest priority to the patient, and asking permission to also address issues of concern to the health coach. Reviewing lab numbers involves assessing the patient’s knowledge about hemoglobin A1c, systolic blood pressure (SBP), or low density lipoprotein (LDL); their most recent results for these measures; the goal for these numbers; and how to reach the goal. In addition to these activities, the health coach takes vital signs and directs the patient to a room.

The health coach stays in the exam room during the medical visit. After the clinician enters the room and speaks with the patient about the reasons for the visit, the health coach may briefly supplement the patient’s summary with information learned during the pre-visit, such as major events since the last visit, agenda items of highest priority to the patient, and issues affecting medication adherence. During the medical visit, the health coach takes notes about the care plan and clinician recommendations. In addition to taking notes on the visit, the health coach may act as an advocate: helping the patient to remember his or her questions and concerns; sharing opportunities for praise, such as actions that the patient is taking to care for his or her health; or alerting the clinician to issues identified during the pre-visit, such as medication not being taken as prescribed.

After the medical visit, the health coach meets with the patient for a post-visit. The post-visit is used to “close the loop” with the patient about the care plan, ensuring that the patient can describe the care plan and recommendations in his or her own words. The health coach is responsible for facilitating navigation of other resources such as diagnostic imaging or referrals to specialists, making follow up appointments, or facilitating introductions to behaviorists or other clinic resources. In addition, the health coach assists the patient in making action plans to increase physical activity, improve healthy eating, reduce stress, or improve medication adherence [36].

In addition to medical visits, the health coach meets with the patient between visits and makes follow-up phone calls between visits. These visits and calls may be used to make action plans or address barriers to carrying out action plans, to assess patient knowledge and share information about target conditions or medication, and to assist with navigation of health and community resources.

### Usual care

Patients randomized to usual care continue to have visits with their clinician over the course of the 12-month period. They have access to any additional resources that are part of usual care at the clinic, including diabetes educators, nutritionists, chronic care nurses, or educational classes.

### Measures

Measures collected through the study include clinical data, patient-reported measures, data abstracted from the medical chart, clinician-reported measures, health coach-reported intervention dose, utilization data, and information for cost analysis.

#### Clinical measures

Hemoglobin A1C, blood pressure, lipids, weight and height are collected at baseline and at 12 months. For diabetic patients, hemoglobin A1c is measured using the DCA Vantage point-of-care testing system. Lipid panels (including calculated LDL) are measured by the clinical laboratory at Clinic A using a Pentra 400 system and through the CardioChek point-of-care testing system at Clinic B. The same method of measurement is used at baseline and at 12 months for each patient. Blood pressure is measured twice, at least two minutes apart, using a calibrated Omron Home Blood Pressure Monitor Model 711-AC on the left arm after the patient has been sitting for at least five minutes. Blood pressure is entered as the average of the two readings unless the two systolic readings differ by more than five points, in which case a third blood pressure reading is taken and the average of all three readings is used. Height is measured using a tape measure and right angle, and weight is measured using a calibrated portable scale.

#### Patient-reported

Surveys at baseline and 12 months examine knowledge of cardiovascular health, chronic disease self-efficacy [37], patient assessment of chronic illness care (PACIC) [38], trust-in-physician [39,40], medication adherence [41,42], proactive behaviors within the medication visit (e.g., asking questions) as measured by an adapted version of the Perceived Efficacy in Patient-Physician Interactions scale (PEPPI) [43], depressive symptoms (PHQ8) [44], the 4-item (short) version of the diabetes distress scale [45,46], physical activity [47-49], visits to the emergency room and hospital, health literacy [50,51], and demographic information. RAs also collect information about prescription medications. At 12 months, patients with a health coach also are queried about interactions with their health coach [38,52,53].

#### Medical chart review

RAs review the patient medical chart soon after baseline and 12 month surveys to abstract the medication list.

#### Clinician-reported

A brief survey at 6–12 months after enrollment of each of their patients in the study examines clinician satisfaction with the patient visit, how the clinician rates the difficulty of the visit, and how well the clinician believes that the patient understood the conversation [54,55]. Qualitative interviews conducted in June–August 2012 examine benefits and challenges of the health coaching model as perceived by the clinicians, as well as recommendations for implementation.

#### Health coach-reported intervention dose

All health coach interactions with patients are documented through a study database to allow estimation of a health coaching “dose” and to identify topics and activities that are most commonly covered during interactions.

#### Utilization of services

The study team is collecting utilization data on clinician, nurse, and nutritionist visits; pharmacy data; and San Francisco Health Plan and SF Department of Public Health Healthy San Francisco claims data on pharmacy medications, emergency room visits and hospitalizations.

### Outcomes

The goal for each of the 3 conditions are defined at 12 month follow-up as: (1) for patients with enrolled with uncontrolled diabetes, a reduction in HbA1c of at least 1.0% from enrollment; (2) for patients enrolled with uncontrolled hypertension, SBP <140 mm Hg (if non-diabetic) or <130 if diabetic; or (3) for patients with uncontrolled hyperlipidemia, LDL <130 mg/dl if non-diabetic or <100 mg/dl if diabetic.

The primary outcome is the proportion of patients in each arm reaching at least one of the above goals. Secondary outcomes are (1) the proportion of patients in each arm meeting each of the goals separately; and (2) the proportion of patients in each arm meeting the composite and separate goals regardless of their level of control at baseline. We will also compare study arms with respect to changes in self-efficacy for chronic disease management, self-care activities, medication adherence, quality of life, depressive symptoms, satisfaction with chronic illness care and health coaching, communication with physician, bed days, emergency room visits and hospitalizations.

### Quality assurance

Data is entered at the time of interview on a dedicated laptop computer into a Microsoft Access database which is programed with skip patterns and range check functions. The project manager reviews consent materials and survey data to identify missing data and collect it soon after baseline. Quality assurance also includes project manager observations of each research assistant and health coach on at least a quarterly basis to ensure adherence to study protocol.

The fidelity of the coaching intervention is supported by the individualized nature of the training which allows for repeated observations of coaching skills until a high level of competency is demonstrated. Prior to working with patients, health coaches conduct a series of videotaped visits with patients and review these with their trainer to improve their coaching skills. In addition, the health coaches meet approximately three times each year for mentoring sessions with study personnel; these sessions provide an opportunity to review and strategize about difficult cases or to seek additional information about health-related subjects.

### Sample size calculation

Sample size and power calculations were performed for the main outcome of interest: the proportion of patients in each arm who achieve a composite goal of 1) decrease of HbA1C by 1% or more; 2) reaching defined blood pressure goal; or 3) reaching defined LDL goal. Based on a review of the literature and examination of data from a similar clinic (the Family Health Center at San Francisco General Hospital) the study team conservatively estimated the proportion of participants achieving the primary outcome to be 25% in the usual care arm and 40% in the coaching arm for an estimated effect size of 15%. To detect this difference, we needed 200 subjects in each arm to achieve a power of .80 using the standard threshold for a significant difference of .05 (2-sided). To account for a 10% loss to follow-up, the total sample size was calculated at 440 patients.

### Data analysis

Initial analyses will compare the frequency of baseline levels of outcome and other key variables (e.g., demographic and disease characteristics) for the intervention and usual care groups using a simple chi-square test for categorical variables and a *t*-test for continuous variables with an approximately normal frequency distribution (with transformation if necessary).

Evaluation of intervention effectiveness will be by intention-to-treat using the above statistical tests. Evidence of effect modification by chronic condition diagnoses will be tested statistically. ANOVA and logistic regression for multivariate analyses will be used to adjust for significant differences identified at baseline between intervention and usual care groups in the outcome analyses. Sensitivity analyses will be performed to estimate the effects of missing data using different assumptions (e.g., imputed values). Additional analyses will be conducted to look for evidence of effect modification by pre-specified subgroups: baseline HbA1c (<9 versus ≥9), SBP (<160 versus ≥160), LDL (< versus ≥ median), language (English primary versus English not primary), and age (<versus ≥ median).

Additionally, a cost analysis of the health coaching intervention will examine utilization data and develop predictive models of cardiovascular events prevented and cost savings. The CORE Diabetes model is a computer model used to determine the long-term health outcomes and economic consequences of interventions in either type 1 or type 2 diabetes mellitus. The model projects outcomes for populations by taking into account (a) baseline cohort characteristics and past history of complications; (b) progression of risk factors and transition probabilities between health states based on published sources; (c) effectiveness of treatments such as current and future diabetes management and concomitant medications, screening strategies, and changes in physiological parameters over time; and (d) direct and indirect costs, discount rates, and quality-of-life data to perform economic analysis. The CORE model calculates development of complications, life expectancy, quality-adjusted life expectancy and total costs within populations.

### Recruitment, enrollment and baseline characteristics of participants

**Figure 1:**
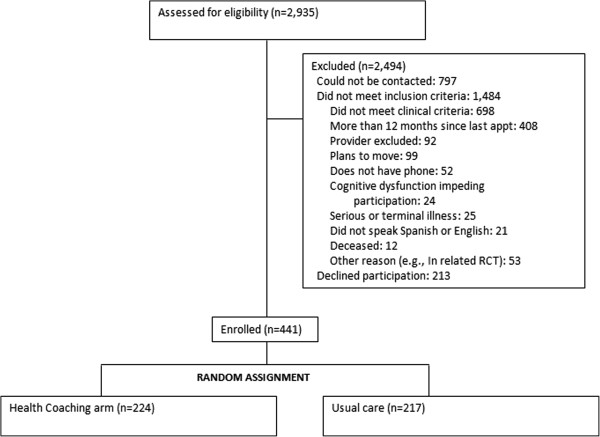
Consort diagram.

A total of 2,935 patients were assessed for eligibility at the two study sites (Figure 1). Most of those screened (2,494) were excluded: 1,484 did not meet inclusion criteria and 797 could not be contacted. Of those who did not meet inclusion criteria, 698 did not meet the clinical criteria because their systolic blood pressure, LDL, and/or hemoglobin A1c were below the thresholds for participation; 408 did not have a medical visits within the preceding 12 months; 99 planned to move; 92 were excluded by a clinician; 52 did not have a phone; and 135 had another reason for exclusion, such a serious or terminal illness. An additional 213 declined participation. The remaining 441 patients were enrolled in the study and randomized to the intervention (n = 224) or usual care arms (n = 217).

**Figure 2:**
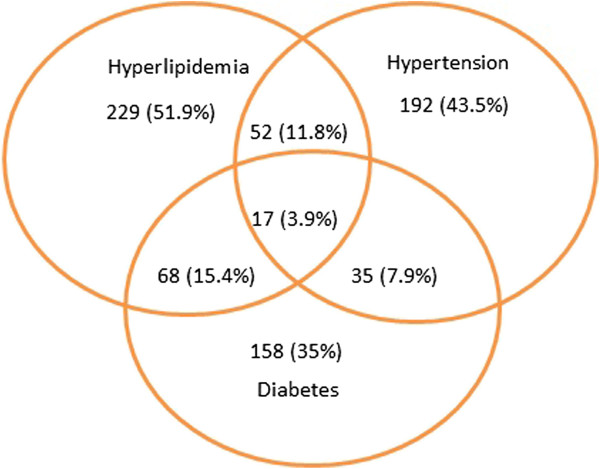
Proportion of participants meeting each of the 3 eligibility criteria.

Of the patients enrolled in the health coaching study, about half (51.9%) meet eligibility criteria based on LDL cholesterol measures, just over a third qualify based on hemoglobin A1c (35.0%), and 43.5% qualify based on systolic blood pressure (Figure 2). More than a third (39.0%) of patients qualify based on more than one measure, and a few (3.9%) qualify based on all three measures.

Compared to patients who declined participation in the health coaching study (n = 213), study participants are more likely to speak Spanish and to attend Clinic A (Table 1). Study participants and people who declined participation do not vary significantly on age or gender.

**Table 1:** Comparison of enrolled patients and refusals

	**Enrolled Mean (SD) or Proportion (number)**	**Refusals Mean (SD) or Proportion (number)**	**Significance**
Age	53.1 (11.1)	54.3 (12.6)	
Gender (female)	57.6% (254)	55.9% (119)	
Language (Spanish)	72.6% (320)	55.9% (119)	p < .001
Clinic (Clinic A)	75.3% (332)	60.6% (129)	p < .001

The mean age of people enrolled in the study is 53 years of age (Table 2). Just over half (55.3%) are female, and 53% report being married or in a long-term relationship. About three-quarters of enrollees are first generation immigrants who were born outside of the United States and 68% speak Spanish as their primary language. Fewer than half (43%) of participants have a high school degree or equivalent; full and part time workers account for 44% of the sample. More than half (58%) of participants in the study report an annual household income of $10,000 or less. Mean body mass index (BMI) was 31, which is in the range of obesity. Mean hemoglobin A1c is 9.9, mean LDL is 147, and mean systolic blood pressure is 159 for patients qualifying for the study based on each of these respective measures.

**Table 2:** Demographic and clinical characteristics of patients enrolled (n = 441)

		**Mean ±SD or Proportion (n)**
**Demographic characteristics**		
Age		52.7 ± 11.1
Gender (female)		55.3% (244)
Currently married or in long term relationship		53.1% (234)
Born in the US		25.6% (113)
Years in US*		18.2 ± 11.1
Primary Language:	English	27.7% (122)
	Spanish	68.7% (303)
	Other	3.6% (16)
Ethnicity:	Asian	4.1% (18)
	African American	19.0% (84)
	Latino or Hispanic	70.1% (309)
	White	2.5% (11)
	Other	4.3% (19)
Working status:	Full time	18.6% (82)
	Part time	25.6% (113)
	Homemaker	13.8% (61)
	Unemployed	16.1% (71)
	Retired	10.0% (44)
	Disabled/SSI	13.6% (60)
	Other	2.3% (10)
Education:	Never went to school	4.3% (19)
	1st to 5th grade	18.4% (81)
	6th to 8th grade	21.1% (93)
	Some high school	13.4% (59)
	High school grad or GED	17.7% (78)
	Some college	15.6% (69)
	College graduate	9.5% (42)
Income:	Less than 5 K	34.0% (150)
	5 K-10 K	24.3% (107)
	10-20 K	29.5% (130)
	20-40 K	10.2% (45)
	More than 40 K	2.0% (9)
Clinic:	Clinic A	75.3% (332)
	Clinic B	24.7% (109)
Total length of coming to clinic for care (years)		8.7 ± 8.1
**Clinical characteristics**		
Body mass index (BMI)		31.4 ± 6.8
Hemoglobin A1c^**^		9.9 ± 1.5
Low-density lipoprotein (LDL)^**^		146.7 ± 34.7
Systolic blood pressure^**^		159.4 ± 15.4

* For the 328 participants born outside the United States.

**Includes only patients qualifying for the study on this measure (n = 158 for hemoglobin A1c, 229 for LDL, and 192 for systolic blood pressure).

## Discussion

Self-management support is an important component of chronic care management, yet many primary care practices do not consistently provide this support due to limitations of training, time, and resources. Medical assistants are an untapped resource to provide this support by virtue of being more linguistically and culturally concordant than clinicians with patients [32]. Moreover, medical assistants are a relatively economical addition to the care team within resource-limited safety net clinics.

To date, few randomized controlled studies have been published on self-management within primary care settings, particularly within the safety net [25]. Early studies on medical assistant health coaching have shown promise, but most are limited by size or methodology [22-25].

In our study, medical assistants, trained and mentored as health coaches, will work for 12 months with patients who have uncontrolled diabetes, hypertension, or hyperlipidemia. They will accompany patients to their medical visits, meeting before and after the visits to ensure that patients voice their questions and leave understanding their care plan. They will work with patients at medical visits and between visits to develop action plans for medication adherence and lifestyle change to improve self-management of their chronic conditions.

Having successfully completed recruitment, this study is uniquely poised to assess the effectiveness of the medical assistant health coaching model in improving clinical outcomes and patient self-management behaviors. Moreover, this study will provide information on barriers and facilitators to implementation, including health coaching’s impact on the clinician experience, and it will examine the business case for the sustainability of this model.

## Abbreviations

HDL: High-density lipoprotein (“good” cholesterol); HbA1c: Hemoglobin A1c (glycosylated hemoglobin); LDL: Low-density lipoprotein (“bad” cholesterol); RA: Research associate; SBP: Systolic blood pressure.

## Competing interests

All of the authors declare that they have competing interests.

## Authors’ contributions

EC, TB, DT, DH, and RWG conceived and directed the study. DT, EC, and DH designed the survey measures and planned the statistical analyses. DD and RWG wrote the study protocol. RWG drafted the manuscript. All authors revised and approved the manuscript.

## Pre-publication history

The pre-publication history for this paper can be accessed here:

http://www.biomedcentral.com/1471-2296/14/27/prepub
